# Prevalence and prognosis of multimorbidity in heart failure with mildly reduced ejection fraction

**DOI:** 10.1007/s00392-025-02840-z

**Published:** 2026-01-19

**Authors:** Marielen Reinhardt, Michael Behnes, Mohammad Abumayyaleh, Thomas Bertsch, Michelle Goertz, Noah Abel, Alexander Schmitt, Felix Lau, Jonas Dudda, Kathrin Weidner, Ibrahim Akin, Tobias Schupp

**Affiliations:** 1 Department of Cardiology, Angiology, Haemostaseology and Medical Intensive Care, University Medical Centre Mannheim, Medical Faculty Mannheim, Heidelberg University, Heidelberg, Germany; 2 Institute of Clinical Chemistry, Laboratory Medicine and Transfusion Medicine, Nuremberg General Hospital, Paracelsus Medical University, Nuremberg, Germany

**Keywords:** Heart failure with mildly reduced ejection fraction, HFmrEF, Multimorbidity, Comorbidities, Mortality

## Abstract

**Background and Objective:**

Related to ongoing demographic, the number of patients with cardiac and non-cardiac comorbidities increases. Heart failure with mildly reduced ejection fraction (HFmrEF) represents a heterogeneous population with diverse clinical profiles. The study investigates the prevalence and prognostic impact of multimorbidity in patients hospitalized with HFmrEF.

**Methods:**

Consecutive patients with HFmrEF were retrospectively included at one institution from 2016 to 2022 and divided into four groups based on the number of concomitant comorbidities taking into account 12 comorbidities (i.e., 0–1, 2–3, 4–5, ≥ 6 comorbidities). The prognostic impact of the number of comorbidities was investigated with regard to the primary endpoint all-cause mortality at 30 months.

**Results:**

From 2,184 patients hospitalized with HFmrEF, 37% presented with 4–5 comorbidities, 17% with ≥ 6 comorbidities. Compared to patients with 4–5, 2–3, 0–1 comorbidities, patients with ≥ 6 comorbidities were more frequently discharged with beta-blockers (83.6% vs. 78.7% vs. 77.6% vs. 64.7%; p = 0.001) and mineralocorticoid receptor antagonists (MRA) (17.2% vs. 15.7% vs. 12.8% vs. 7.9%; p = 0.004). The risk of all-cause mortality at 30 months was higher in patients with ≥ 6 comorbidities compared to patients with less comorbidities (i.e., 4–5, 2–3, 0–1) (59.5% vs. 38.6% vs. 17.0% vs. 7.9%, p = 0.001). Both cardiovascular (HR = 1.106; 95% CI 1.030 – 1.188; p = 0.006) and non-cardiovascular (HR = 1.564; 95% CI 1.470 – 1.664; p = 0.001) comorbidities predicted the risk of long-term all-cause mortality.

**Conclusion:**

In patients hospitalized with HFmrEF, more than 50% had at least 4 comorbidities. Both cardiovascular and non-cardiovascular comorbidities predicted the risk of long-term all-cause mortality in HFmrEF.

**Supplementary Information:**

The online version contains supplementary material available at 10.1007/s00392-025-02840-z.

## Lay Summary

In patients hospitalized with HFmrEF, multimorbidity is common and associated with poor prognosis.

The three most frequent comorbidities were arterial hypertension, anemia, and atrial fibrillation/flutter.

The risk of mortality was higher with an increasing number of comorbidities and the event rates for non-cardiovascular comorbidities were higher than for cardiovascular comorbidities.

**Graphical Abstract:**

Prevalence and prognosis of multimorbidity in HFmrEF. AF, atrial fibrillation; HFmrEF, heart failure with mildly reduced ejection fraction.

**Supplementary Information:**

The online version contains supplementary material available at 10.1007/s00392-025-02840-z.

## Graphical abstract

**Figure:**
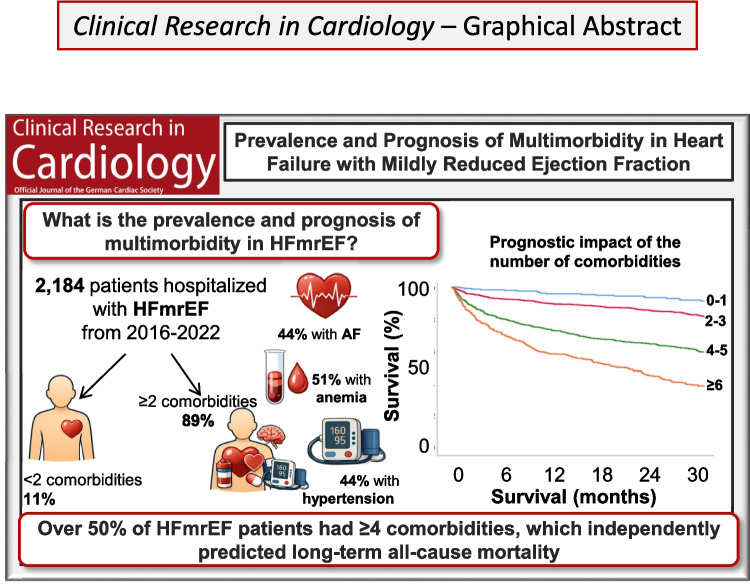

## Introduction

Heart failure (HF) affects more than 64 million people worldwide and is accompanied by a 1-year mortality rate of 15–30% [1]. Due to demographic changes and an ageing population, HF patients have become more complex, facing a multifactorial disease worsened by cardiovascular and non-cardiovascular comorbidities [2, 3]. Multimorbidity, defined as the coexistence of at least two chronic conditions [4], is now the norm, especially among elderly patients [5]. Given the increasing prevalence, multimorbidity has considerably gained prominence in clinical practice [6]. Consequently, both US and European guidelines for the management of HF emphasize the identification and treatment of concomitant comorbidities to provide best medical care and potentially reduce adverse outcomes [7, 8]. Beyond worsening prognosis and quality of life, multimorbidity poses a considerable economic and financial burden both individually and globally [9, 10]. Accordingly, research has shifted from a single-disease framework where most studies excluded individuals with multiple conditions to a more holistic approach including incident and prevalent comorbidities [5, 11].

HF rarely occurs in isolation, with about 40% of HF patients being accompained by more than five comorbidities and over 80% accompanied by at least two comorbidities [12–16]. These include both cardiovascular and non-cardiovascular comorbidities, such as obesity, chronic kidney disease, diabetes mellitus, hypertension, and atrial fibrillation [3, 17]. While the most common comorbidities in HF are cardiovascular related, non-cardiovascular causes may be linked to even higher hospitalization and mortality rates [18, 19]. Moreover, symptoms in patients with HF are dominated by comorbidities and significantly impact health status, especially when they are persisting and quality of life remains poor even with optimized HF treatment [20, 21]. Overall, multimorbidity increases event rates in both chronic [22, 23] and acute [24, 25] HF, the risk of mortality and hospitalization [13]. However, a higher comorbidity burden implies an increased complexity of clinical management and suboptimal guideline directed medical treatment (GDMT) [24]. Despite the adverse outcome of multimorbidity, combined use of HF drugs decreases alongside the comorbidity burden, although GDMT was shown to be beneficial regardless of the number of comorbidities [13, 26].

The HF population is complex and heterogeneous, with significant differences depending on the phenotype of the left ventricular ejection fraction (LVEF) with regard to sex, age, outcomes and comorbidities [27]. While the prevalence of both cardiovascular and non-cardiovascular comorbidities is high in all HF subtypes, patients with HF and preserved ejection fraction (HFpEF) were shown to have the highest burden of comorbidities [13]. Recent studies have researched the prevalence and implications of multimorbidity in HF across LVEF groups, however data concerning the phenotype of HF with mildly reduced EF (HFmrEF) mostly relies on post-hoc analyses from pre-existing trials and registries [28]. HFmrEF represents a heterogeneous and intermediate entity within the HF spectrum. It shares the higher prevalence of ischemic heart disease and the sex distribution (more males than females) with HFrEF, while overlapping with HFpEF especially in terms of non-cardiac comorbidities such as hypertension, atrial fibrillation and obesity [29]. These mixed pathophysiological features contribute to varied clinical presentations, underscoring the importance of a more refined characterization to define HFmrEF as a distinct clinical entity.

To achieve better quality of life and provide specific data for this newly introduced patient group, this study investigates the relationship between comorbidities and outcomes in HFmrEF using a large retrospective data-set.

## Methods

### Study patients, design and data collection

For the present study, all patients hospitalized with HFmrEF at one University Medical Centre between January 2016 and December 2022 were retrospectively included [30]. Using the electronic hospital information system, all relevant clinical data related to the index event were documented, as previously published [31].

The present study derived from a retrospective single-centre all-comers registry including consecutive patients with HFmrEF hospitalized at the University Medical Centre Mannheim (UMM), Germany (clinicaltrials.gov identifier: NCT05603390), based on the “Heart Failure With Mildly Reduced Ejection Fraction” (HARMER) Registry. The registry was carried out according to the principles of the declaration of Helsinki and was approved by the medical ethics committee II of the Medical Faculty Mannheim, University of Heidelberg, Germany (ethical approval code: 2022–818, approval date 4 April 2022).

### Inclusion and exclusion criteria

The present study included all patients aged ≥ 18 years hospitalized with HFmrEF at one institution. All patients underwent at least one standardized transthoracic echocardiography during their index hospitalization, conducted by cardiologists as part of routine clinical care and in line with current European guidelines [32]. According to the “2021 European Society of Cardiology (ESC) guidelines for the diagnosis and treatment of acute and chronic HF” the diagnosis of HFmrEF was determined and all patients with LVEF 41–49% and symptoms and/or signs of HF were included [8]. From a total of 2,228 patients with HFmrEF, 44 patients with no evidence on long-term follow-up were excluded (corresponding lost-to follow-up rate: 1.97%).

### Risk stratification and definitions of comorbidities

For the present study a total of 12 comorbidities were considered and defined through clinical routine after review of available medical charts. Non cardiovascular comorbidities included anemia (defined as haemoglobin levels < 12 g/dL in female patients and < 13 g/dL in male patients),diabetes mellitus (defined by prior or current HbA1c ≥ 6,5%), chronic kidney disease (CKD) (defined as estimated glomerular filtration rate (eGFR) < 60 ml/min/1.73m^2^), prior/current malignancy, liver cirrhosis, and chronic obstructive pulmonary disease (defined in accordance with the GOLD Initiative [33]). Cardiovascular comorbidities comprised valvular heart disease (including moderate or severe aortic valve stenosis, aortic regurgitation, mitral regurgitation and/or tricuspid regurgitation, identified by transthoracic or transesophageal echocardiography prior or during hospitalization index), arterial hypertension (defined as systolic blood pressure ≥ 140 mmHg and/or diastolic blood pressure ≥ 90 mmHg), atrial fibrillation/flutter, prior/current acute myocardial infarction (AMI), prior/current stroke and peripheral artery disease. Patients were then stratified into four groups based on the number of concomitant comorbidities: 0–1, 2–3, 4–5 and ≥ 6 comorbidities, further risk stratification was performed according to the overall number of comorbidities (per 1 comorbidity increase) and stratified by cardiovascular and non-cardiovascular comorbidities. Multimorbidity was defined according to the World Health Organization definition as the coexistence of 2 or more chronic comorbidities in one individual (Multimorbidity: Technical Series on Safer Primary Care. Geneva: World Health Organization; 2016. Licence: CC BY-NC-SA 3.0 IGO).

### Study endpoints

The primary endpoint was all-cause mortality during median follow-up of 30 months. Secondary endpoints comprised in-hospital all-cause, cardiac and non-cardiac mortality, all-cause mortality at 12 months, rehospitalization for worsening HF at 12 and 30 months, cardiac rehospitalization, AMI, stroke, coronary revascularization and major adverse cardiac and cerebrovascular events (MACCE) during follow-up.

 HF-related hospitalization was defined as a rehospitalization due to worsening HF requiring intravenous diuretic therapy. HF-related rehospitalization comprised patients with hospitalization due to worsening HF as the primary cause or as a result of another cause but associated with worsening HF at the time of admission, or as a result of another cause but complicated by worsening HF during its cause. Cardiac rehospitalization was defined as rehospitalization due to a primary cardiac condition, including worsening HF, AMI, coronary revascularization and symptomatic atrial or ventricular arrhythmias. MACCE was defined as composite of all-cause mortality, coronary revascularization, non-fatal AMI and non-fatal stroke.

### Statistical methods

Quantitative data were shown as median and interquartile range (IQR), and ranged depending on the distribution of the data. They were compared using the Kruskal–Wallis-Test. The Kolmogorov–Smirnov test was used to assess deviations from a Gaussian distribution. Qualitative data were presented as absolute and relative frequencies and were compared using the chi-square test or the Fisher’s exact test, as appropriate. Kaplan–Meier analyses were performed stratified by the number of comorbidities and univariable hazard ratios (HR) were given together with 95% confidence intervals (CIs). Uni- and multivariable Cox regression analyses were performed within the entire study cohort and adjusted for age, sex and the number of comorbidities, as well as stratified by cardiovascular and non-cardiovascular comorbidities (per 1 comorbidity increase). Multivariable Cox regression analyses were visualized using forest plots. Multivariate analyses were restricted to the primary endpoint and HF-related rehospitalization due to event numbers and to avoid model overfitting; secondary endpoints were therefore analyzed using univariate methods only. Proportional hazard assumptions were not tested.

Results of all statistical tests were considered significant for p ≤ 0.05. SPSS (Version 28, IBM, Armonk, New York) was used for statistics.

## Results

### Study population

**Fig. 1:**
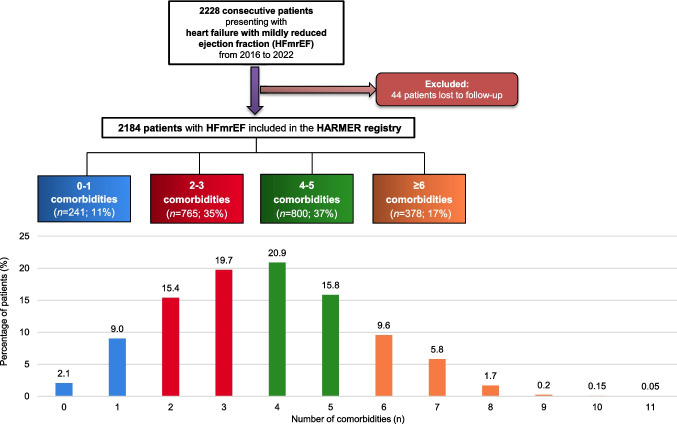
Flow chart of the study population

**Fig. 2:**
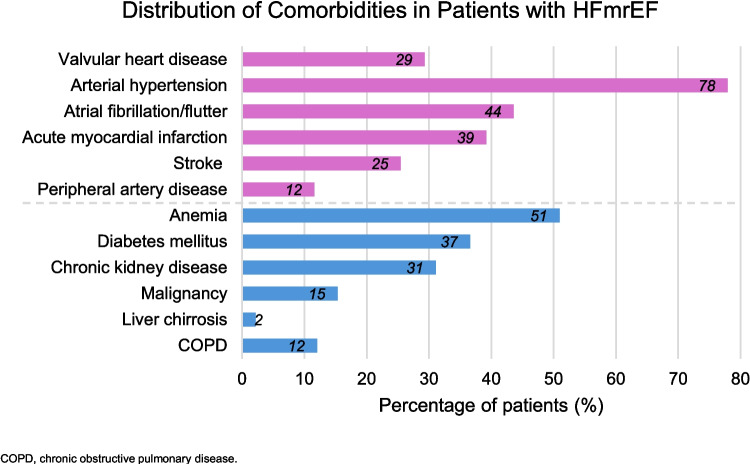
Distribution of comorbidities in patients with HFmrEF

From 2016 to 2022, a total of 2,228 consecutive patients with HFmrEF were hospitalized at our institution. 44 patients (1.97%) were lost to follow-up and excluded from the study. From a total of 2,184 patients included in the registry, 241 (11%) patients presented with 0–1, 765 (35%) with 2–3, 800 (37%) with 4–5 and 378 (17%) with ≥ 6 comorbidities (Fig. 1). The most frequent comorbidities were arterial hypertension (78%), followed by anemia (51%), atrial fibrillation/flutter (44%), prior/current AMI (39%), diabetes mellitus (37%), CKD (31%), valvular heart disease (29%), stroke (25%), malignancy (15%), peripheral artery disease (12%) and chronic obstructive pulmonary disease (COPD) (12%), liver cirrhosis (2%) (Fig. 2).

Baseline characteristics of the overall HARMER population are showed in supplementary Table 1. Patients with ≥ 6 comorbidities were older compared to patients with 4–5, 3–2, 1–0 comorbidities (median 81 vs. 78 vs. 71 vs. 57 years; p = 0.001), whereas no difference was found between sex distribution (p = 0.064) (Table 1). Patients with ≥ 6 comorbidities had the highest rates of coronary artery disease (CAD) (65.3%), prior congestive HF (57.1%) and acute decompensated HF in the last 12 months before index hospitalization (20.4%). No significant differences regarding prior LVEF were found across the analysed subgroups. Throughout all comorbidity groups, ischemic cardiomyopathy (ICM) was the most frequent HF aetiology, however higher rates were shown in patients with ≥ 6 comorbidities compared to patients with 4–5, 2–3, 0–1 comorbidities (73.5% vs. 59.4% vs. 53.9% vs. 38.6%; p = 0.001) (Supplementary Table 2). On the other hand, patients with 0–1 comorbidities had the highest rates of non-ischemic cardiomyopathy (NICM) compared to patients with 2–3, 4–5 and ≥ 6 comorbidities (13.3% vs. 6.8% vs. 5.1% vs. 6.3%; p = 0.001). Regarding relevant echocardiographic data, patients with 0–1 comorbidities had better right ventricular function (median tricuspid annular plane systolic excursion (TAPSE) 21 vs. 19 mm; p = 0.001) and less frequently presented with diastolic dysfunction (58.9% vs. 73.0%; p = 0.001) than patients with ≥ 6 comorbidities. A higher proportion of patients with 2–3 comorbidities underwent coronary angiography (49.0% vs. 49.4% vs. 35.4% vs. 32.0%, for patients with 0–1, 2–3, 4–5, ≥ 6 comorbidities respectively; p = 0.001) but the highest rates of 3-vessel CAD were found in patients with ≥ 6 comorbidities (62.8% vs. 46.6% vs. 36.2% vs. 18.6%, for patients with ≥ 6, 4–5, 2–3, 0–1 comorbidities respectively; p = 0.001). Among relevant laboratory values, patients with ≥ 6 comorbidities had higher creatinine levels (median 1.62 vs. 1.15 vs. 0.96 vs. 0.89 mg/dL; p = 0.001) and lower eGFR (median 40 vs. 58 vs. 76 vs. 89 mL/min/1.73m^2^; p = 0.001) compared to patients with 4–5, 2–3 and 0–1 comorbidities. Furthermore, N-terminal pro-B-type natriuretic peptide (NT-pro-BNP) levels were higher in patients with ≥ 6 comorbidities (median 6410 vs. 3758 vs. 1590 vs. 735 pg/mL; p = 0.001).

**Table 1:** Baseline characteristics

	0–1 Comorbidities (*n* = 241)		2–3 Comorbidities (*n* = 765)		4–5 Comorbidities (*n* = 800)		≥ 6 Comorbidities (*n* = 378)		*p* value
Age, median (IQR)	57 (45–70)		71 (61–80)		78 (70–84)		81 (75–85)		**0.001**
Male sex, *n* (%)	165	(68.5)	502	(65.6)	489	(61.1)	254	(67.2)	0.064
Body mass index, kg/m^2^, median (IQR)	26.4 (24.2–29.7)		27.2 (24.1–31.2)		26.3 (23.6–30.1)		26.2 (23.6–30.0)		**0.042**
SBP, mmHg, median (IQR)	140 (128–158)		144 (128–164)		144 (123–167)		140 (120–159)		**0.026**
DBP, mmHg, median (IQR)	80 (70–94)		81 (72–93)		78 (67–90)		71 (63–84)		**0.001**
Heart rate, bpm, median (IQR)	82 (70–95)		81 (70–96)		80 (68–94)		78 (66–93)		0.055
Medical history, *n* (%)									
Coronary artery disease	34	(14.1)	234	(30.6)	381	(47.6)	247	(65.3)	**0.001**
Prior myocardial infarction	13	(5.4)	120	(15.7)	221	(27.6)	167	(44.2)	**0.001**
Prior PCI	26	(10.8)	170	(22.2)	249	(31.1)	167	(44.2)	**0.001**
Prior CABG	5	(2.1)	37	(4.8)	100	(12.5)	72	(19.0)	**0.001**
Prior valvular surgery	9	(3.7)	26	(34.)	38	(4.8)	23	(6.1)	0.182
Prior congestive heart failure	38	(15.8)	186	(24.3)	301	(37.6)	216	(57.1)	**0.001**
Prior LVEF ≥ 50, *n* (%)	10	(37.0)	55	(45.8)	89	(47.5)	78	(55.3)	0.219
Prior LVEF 41–49%, *n* (%)	13	(48.2)	44	(36.7)	62	(33.2)	43	(30.5)	0.307
Prior LVEF ≤ 40, *n* (%)	4	(14.8)	21	(17.5)	36	(19.3)	20	(14.2)	0.666
Prior LVEF not documented, *n* (%)	11	(28.9)	66	(35.5)	114	(37.9)	75	(34.7)	
Decompensated heart failure < 12 months	9	(3.7)	55	(7.2)	97	(12.1)	77	(20.4)	**0.001**
Prior ICD	1	(0.4)	20	(2.6)	14	(1.8)	7	(1.9)	0.173
Prior sICD	3	(1.2)	1	(0.1)	2	(0.3)	3	(0.8)	0.060
Prior CRT-D	3	(1.2)	9	(1.2)	10	(1.3)	10	(2.6)	0.219
Prior Pacemaker	10	(4.1)	37	(4.8)	91	(11.4)	62	(16.4)	**0.001**
Chronic kidney disease	1	(0.4)	84	(11.0)	308	(38.5)	286	(75.7)	**0.001**
Peripheral artery disease	1	(0.4)	21	(2.7)	94	(11.8)	137	(36.2)	**0.001**
Stroke	3	(1.2)	68	(8.9)	148	(18.5)	112	(29.6)	**0.001**
Liver cirrhosis	0	(0.0)	3	(0.4)	17	(2.1)	27	(7.1)	**0.001**
Malignancy	5	(2.1)	90	(11.8)	136	(17.0)	104	(27.5)	**0.001**
COPD	2	(0.8)	32	(4.2)	114	(14.2)	115	(30.4)	**0.001**
Cardiovascular risk factors, *n* (%)									
Arterial hypertension	53	(22.0)	561	(73.3)	726	(90.8)	362	(95.8)	**0.001**
Diabetes mellitus	6	(2.5)	174	(22.7)	363	(45.4)	256	(67.7)	**0.001**
Hyperlipidemia	45	(18.7)	215	(28.1)	255	(31.9)	147	(38.9)	**0.001**
Smoking	104	(43.2)	283	(37.0)	260	(32.5)	149	(39.4)	**0.009**
Current	76	(31.5)	165	(21.6)	120	(15.0)	45	(11.9)	**0.001**
Former	28	(11.6)	118	(15.4)	140	(17.5)	104	(27.5)	**0.001**
Family history	40	(16.6)	90	(11.8)	48	(6.0)	23	(6.1)	**0.001**
Comorbidities at index hospitalization, n (%)									
Unstable angina	15	(6.2)	43	(5.6)	35	(4.4)	6	(1.6)	**0.010**
STEMI	30	(12.4)	83	(10.8)	49	(6.1)	14	(3.7)	**0.001**
NSTEMI	19	(7.9)	93	(12.2)	94	(11.8)	68	(18.0)	**0.001**
Acute decompensated heart failure	18	(7.5)	94	(12.3)	221	(27.6)	151	(39.9)	**0.001**
Cardiogenic shock	6	(2.5)	14	(1.8)	18	(2.3)	15	(4.0)	0.168
Atrial fibrillation	19	(7.9)	226	(29.5)	409	(51.1)	262	(69.3)	**0.001**
Atrial flutter	5	(2.1)	13	(1.7)	13	(1.6)	5	(1.3)	0.912
Cardiopulmonary resuscitation	7	(2.9)	16	(2.1)	17	(2.1)	13	(3.4)	0.468
Out-of-hospital	4	(1.7)	8	(1.0)	6	(0.8)	4	(1.1)	0.664
In-hospital	3	(1.2)	8	(1.0)	11	(1.4)	9	(2.4)	0.344
Stroke	16	(6.6)	101	(13.2)	130	(16.3)	51	(13.5)	**0.002**
Medication at index admission, *n* (%)									
ACE-inhibitor	35	(14.5)	249	(32.5)	339	(42.4)	152	(40.2)	**0.001**
ARB	20	(8.3)	156	(20.4)	204	(25.5)	109	(28.8)	**0.001**
Beta-blocker	57	(23.7)	359	(46.)	523	(65.4)	295	(78.0)	**0.001**
Aldosterone antagonist	9	(3.7)	53	(6.9)	91	(11.4)	53	(14.0)	**0.001**
ARNI	2	(0.8)	7	(0.9)	8	(1.0)	2	0.5)	0.876
SGLT2-inhibitor	0	(0.0)	17	(2.1)	17	(2.1)	11	(2.9)	0.088
Loop diuretics	13	(5.4)	169	(22.1)	368	(46.0)	271	(71.1)	**0.001**
Statin	45	(18.7)	265	(34.6)	423	(52.9)	252	(66.7)	**0.001**
ASA	48	(19.9)	254	(33.2)	294	(36.8)	139	(36.8)	**0.001**
P2Y12-inhibitor	10	(4.1)	60	(7.8)	86	(10.8)	55	(14.6)	**0.001**
DOAC	21	(8.7)	123	(16.1)	238	(29.8)	138	(36.5)	**0.001**
Vitamin K antagonist	4	(1.7)	51	(6.7)	75	(9.4)	55	(14.6)	**0.001**

*ACEi*, angiotensin-converting-enzyme inhibitor; *ARB*, angiotensin receptor blocker; *ARNI*, angiotensin receptor neprilysin inhibitor; *ASA*, acetylsalicylic acid; *CABG*, coronary artery bypass grafting; *COPD*, chronic obstructive pulmonary disease; *CRT-D*, cardiac resynchronization therapy with defibrillator; *DBP*, diastolic blood pressure; *DOAC*, directly acting oral anticoagulant; *ICD*, implantable cardioverter defibrillator; *IQR*, interquartile range; *(N)STEMI*, non-ST-segment elevation myocardial infarction; *SBP*, systolic blood pressure; *SGLT2*, sodium glucose linked transporter 2; *s-ICD*, subcutaneous implantable cardioverter defibrillator

Level of significance *p* ≤ 0.05. Bold type indicates statistical significance

### Distribution of comorbidities stratified by age in patients with HFmrEF

A trend of increasing number of comorbidities with increasing age was observed throughout all groups (Supplementary Table 3). While in the 30th, 40th and 50th decades, most patients had 0–1 comorbidities (i.e., respectively, 60.0%, 60.0% and 47.8%), patients aged from 50 to 59 and 60 to 69 most frequently presented 2–3 comorbidities (i.e., respectively, 49.8% vs. 44.3%) and among patients in the 80th, 90th and 100th decades most patients had 4–5 comorbidities (i.e., respectively, 38.8%, 46.2%, 51.4%). The presence of ≥ 6 comorbidities was not reported in patients aged from 20 to 49 years and increased with increasing age (3.7% in the 60th, 11.1% in the 70th, 20.6% in the 80th, 25.0% in the 90th, and 30.3% in the 100th decade).

### Treatment across comorbidities groups

Treatment rates of renin–angiotensin–aldosterone-system (RAAS) inhibitors were highest in patients with 4–5 comorbidities compared to patients with 0–1, 2–3 and ≥ 6 comorbidities (76.3% vs. 61.8% vs. 75.9% vs. 71.3%; p = 0.001) (Supplementary Fig. 1). Patients with ≥ 6 comorbidities more frequently received beta-blockers (83.6% vs. 78.7% vs. 77.6% vs. 64.7%; p = 0.001) and mineralocorticoid receptor blockers (MRA) (17.2% vs. 15.7% vs. 12.8% vs. 7.9%; p = 0.004) compared to patients with less comorbidities (i.e., 4–5, 3–2, 0–1). The rates of angiotensin receptor-neprilysin inhibitor (ARNI) and sodium glucose linked transporter 2 (SGLT2)-inhibitors were low across all groups, and no significant difference for the treatment with ARNI (p = 0.807) and SGLT2-inhibitors (p = 0.335) were found across all groups. Furthermore, patients with ≥ 6 comorbidities were more likely to receive treatment with loop diuretics (83.3% vs. 58.6% vs. 32.3% vs. 14.9%; p = 0.001).

### Prognostic impact of multimorbidity in patients with HFmrEF

**Fig. 3:**
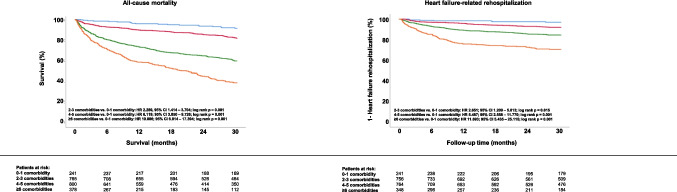
Kaplan–Meier analyses demonstrating the prognostic impact of comorbidities on the risk of all-cause mortality (left panel) and hospitalization for worsening HF (right panel) in patients with HFmrEF stratified by four comorbidity groups (i.e. 0–1, 2–3, 4–5, ≥ 6)

The primary endpoint all-cause mortality at 30 months occurred in 7.9% of patients with 0–1, 17.0% of patients with 2–3, 38.6% of patients with 4–5 and 59.5% of patients with ≥ 6 comorbidities (p = 0.001) (Table 2). Accordingly, the risk of all-cause mortality increased with the number of comorbidities and was highest for patients with ≥ 6 comorbidities compared to patients with 0–1 comorbidities (HR = 10.886; 95% CI 6.814–17.394; log rank p = 0.001) (Fig. 3; left panel). Accordingly, the risk of HF-related rehospitalization at 30 months was higher in patients ≥ 6 comorbidities compared to patients with less comorbidities (i.e., 4–5, 2–3, 0–1) (29.0% vs. 14.9% vs. 7.5% vs. 2.9%; p = 0.001) with a more than 11-fold risk increase compared to patients with 0–1 comorbidities (HR = 11.693; 95% CI 5.435–25.115; log rank p = 0.001) (Fig. 3; right panel). Both the rates of cardiac (3.4% vs. 0.8% vs. 0.7% vs. 0.0%; p = 0.001) and non-cardiac (4.5% vs. 3.8% vs. 0.5% vs. 0.0%; p = 0.001) in-hospital mortality were higher in patients with ≥ 6 comorbidities compared to patients with 4–5, 2–3 and 0–1 comorbidities. Furthermore, compared to patients with 4–5, 2–3 and 0–1 comorbidities, patients with ≥ 6 comorbidities showed higher rates of cardiac rehospitalization (33.3% vs. 23.7% vs. 18.1% vs. 11.6%; p = 0.001) and MACCE (63.5% vs. 46.4% vs. 25.4% vs. 14.9%; p = 0.001), whereas no significant difference in the rates of coronary revascularization during follow-up was found among the groups (p = 0.942). Finally, the duration of hospitalization in days was significantly higher for patients with ≥ 6 comorbidities compared to patients with less comorbidities (i.e., 4–5, 3–2, 0–1) (median days 13 vs. 9 vs. 8 vs. 6; p = 0.001).

**Table 2:** Follow-up data, primary and secondary endpoints

	0–1 Comorbidities (*n* = 241)		2–3 Comorbidities (*n* = 765)		4–5 Comorbidities (*n* = 800)		≥ 6 Comorbidities (*n* = 378)		*p* value
Primary endpoint, *n* (%)									
All-cause mortality, at 30 months	19	(7.9)	130	(17.0)	309	(38.6)	225	(59.5)	**0.001**
Secondary endpoints, *n* (%)									
All-cause mortality, in-hospital	0	(0.0)	9	(1.2)	36	(4.5)	30	(7.9)	**0.001**
Cardiac mortality, in-hospital	0	(0.0)	5	(0.7)	6	(0.8)	13	(3.4)	**0.001**
Non-cardiac mortality, in-hospital	0	(0.0)	4	(0.5)	30	(3.8)	17	(4.5)	**0.001**
All-cause mortality, at 12 months	10	(4.1)	80	(10.5)	218	(27.3)	158	(41.8)	**0.001**
Heart failure-related rehospitalization, at 30 months	7	(2.9)	57	(7.5)	114	(14.9)	101	(29.0)	**0.001**
Heart failure-related rehospitalization, at 12 months	4	(1.7)	32	(4.2)	83	(10.9)	85	(24.4)	**0.001**
Cardiac rehospitalization, at 30 months	28	(11.6)	137	(18.1)	181	(23.7)	116	(33.3)	**0.001**
Coronary revascularization, at 30 months	14	(5.8)	51	(6.7)	53	(6.9)	24	(6.9)	0.942
Acute myocardial infarction, at 30 months	5	(2.1)	18	(2.4)	29	(3.8)	12	(3.4)	0.313
Stroke, at 30 months	3	(1.2)	22	(2.9)	24	(3.1)	8	(2.3)	0.416
MACCE, at 30 months	36	(14.9)	194	(25.4)	371	(46.4)	240	(63.5)	**0.001**
Follow-up data, median (IQR)									
Hospitalization time, days	6 (4–10)		8 (5–12)		9 (6–17)		13 (8–20)		**0.001**
ICU time, days	0 (0–1)		0 (0–1)		0 (0–1)		0 (0–1)		0.489
Follow-up time, days	1428 (800–2048)		1166 (627–1866)		759 (280–1421)		504 (135–991)		**0.001**

ICU, intensive care unit; MACCE, major adverse cardiac and cerebrovascular events. Level of significance p ≤ 0.05. Bold type indicates statistical significance

### Prognostic impact stratified by single comorbidities

**Fig. 4:**
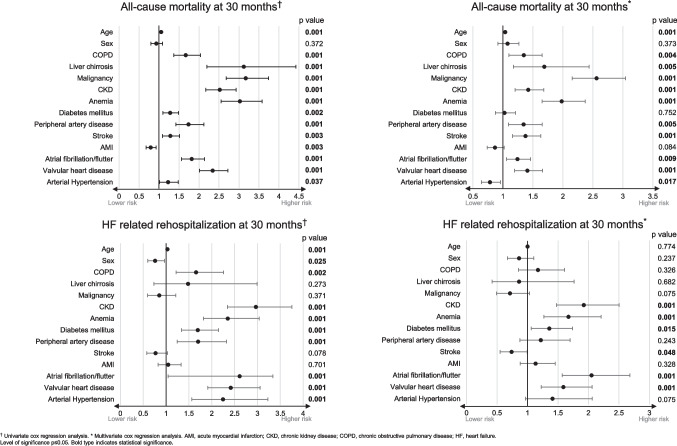
Forest plots demonstrating the risk of all-cause mortality (left upper panel) and HF-related rehospitalization (left lower panel) stratified by single comorbidities after univariate cox regression analyses, as well as the risk of all-cause mortality (right upper panel) and HF-related rehospitalization (right lower panel) stratified by single comorbidities after multivariate cox regression analyses

Cox regression analyses were performed to analyze the impact of each comorbidity. In univariate regression analyses the highest risk for all-cause mortality was registered in patients with prior or current malignancies (HR = 3.168; 95% CI 2.683–3.740; p = 0.001), liver cirrhosis (HR = 3.118; 95% CI 2.197–4.425, p = 0.001) and anemia (HR = 3.021; 95% CI 2.551–3.576; p = 0.001) (Fig. 4; upper left panel). The risk of HF related rehospitalization was highest in patients with CKD (HR = 2.966; 95% CI 2.343–3.755; p = 0.001), atrial fibrillation (HR = 2.611; 95%% CI 1.043–3.337; p = 0.001) and valvular heart disease (HR = 2.417; 95% CI 1.911–3.058; p = 0.001) (Fig. 4; upper right panel).

The highest risk for all-cause mortality was in patients with prior or current malignancies (HR = 2.561; 95% CI 2.155–3.044; p = 0.001), followed by anemia (HR = 1.979; 95% CI 1.652–2.371; p = 0.001) and liver cirrhosis (HR = 1.690; 95% CI 1.173–2.435; p = 0.005) (Fig. 4; lower left panel). Atrial fibrillation (HR = 2.051; 95% CI 1.570–2.680; p = 0.001), CKD (HR = 1.923; 95% CI 1.477–2.502; p = 0.001) and anemia (HR = 1.673; 95% CI 1.269–2.207; p = 0.001) were associated with the highest risk of HF related rehospitalization (Fig. 4; lower right panel).

### Prognostic impact of multimorbidity according to the number of comorbidities

Even after stratifying patients into 9 groups based on the number of comorbidities, a higher number of comorbidities remained associated with an increasing risk of all-cause mortality, the rates of mortality being lowest for patients with 0 comorbidities (6.6%) and highest for patients with at least 8 comorbidities (77.2%) (log rank p = 0.001) (Supplementary Fig. 2; left panel).

### Prognostic impact of multimorbidity after multivariable adjustment

After multivariable adjustment, increasing age was shown to significantly affect the risk of all-cause mortality for patients with HFmrEF (HR = 1.037; 95% CI 1.029–1.045; p = 0.001), but not the risk of HF-related rehospitalization at 30 months (HR 1.010; 95% CI 0.999–1.022; p = 0.086) (Supplementary Fig. 3; left upper and lower panels). On the contrary, sex did not affect the risk of all-cause mortality (p = 0.312) or HF-related rehospitalization (p = 0.052). Furthermore, even after multivariable adjustment, a higher number of comorbidities was associated with a significantly higher risk of all-cause mortality (HR for patients with ≥ 6 comorbidities compared to patients with 0–1 comorbidity: 5.793; 95% CI 3.577–9.382; p = 0.001) and HF-related rehospitalization (HR 9.505; 95% CI 4.279–21.112; p = 0.001). Finally, the risk of all-cause mortality (HR = 1.295; 95% CI 0.377–4.444; p = 0.673) and HF-related rehospitalization (HR = 1.409; 95% CI 0.170–11.780; p = 0.741) was not significantly higher in patients with 1 comorbidity as compared to 0 comorbidities.

Additionally, both non-cardiovascular and cardiovascular comorbidities were associated with an increased risk of all-cause mortality, with a higher risk for non-cardiovascular than for cardiovascular comorbidities, i.e. respectively 56.4% (95% CI 1.470–1.664; p = 0.001) and 10.6% (95% CI 1.030–1.188; p = 0.006) per comorbidity increase (Supplementary Fig. 3; right upper panel). Accordingly, the risk of rehospitalization for worsening HF was higher for both non-cardiovascular (HR = 1.419; 95% CI 1.287–1.565; p = 0.001) and cardiovascular comorbidities (HR = 1.367; 95% CI 1.226–1.524; p = 0.001) (Supplementary Fig. 3; right lower panel). Finally, male sex was associated with a lower risk of HF-related rehospitalization (HR = 0.770; 95% CI 0.605–0.981; p = 0.035).

## Discussion

This study retrospectively investigated the prevalence of multimorbidity in patients hospitalized with HFmrEF and its impact on the prognosis taking into account 12 common comorbidities. The main findings show that more than 50% of the included patients presented with at least 4 comorbidities, specifically arterial hypertension, anemia, and atrial fibrillation/flutter. Patients with ≥ 6 comorbidities were more frequently discharged with beta-blockers, MRA and loop diuretics. Overall, the risk of mortality was higher with an increasing number of comorbidities, as the primary endpoint all-cause mortality after 30 months occurred in more than half of the patients with ≥ 6 comorbidities. Furthermore, the event rates for non-cardiovascular comorbidities were higher than for cardiovascular comorbidities.

Our findings align with data from different HF studies including European [11, 13, 15], US [14, 18] and Asian [12, 34] populations, where approximately more than half of the included patients presented with at least 4 or 5 comorbidities. More specifically, we found that about 98% of HFmrEF patients had at least 1 comorbidity, which is consistent with data from the Swedish HF (SwedeHF) Registry, where the distribution of comorbidities was analysed across LVEF spectrum and almost 99% of patients with HFmrEF showed at least one comorbidity [13]. In a pooled analysis from the “Prospective Comparison of ARNI with ACEI Inhibitor to Determine Impact on Global Mortality and Morbidity in Heart Failure” (PARADIGM-HF) and “Aliskiren Trial to Minimize Outcomes in Patients with Heart Failure” (ATMOSPHERE) trials [35] the proportion of patients with at least one comorbidity was slightly lower at about 94%, which may be attributable to a broader representation of real-world populations in all-comers registries compared to clinical trials with extensive patient pre-selection.

The prevalence and distribution of comorbidities in HF is known to be different according to the LVEF [29]. Especially HFpEF is associated with a higher burden of comorbidities, as demonstrated in a pooled analysis of data deriving from HFrEF and HFpEF studies [36]. Overall, the prevalence of cardiovascular and non-cardiovascular comorbidities in HFmrEF seems to be intermediate between HFrEF and HFpEF, but data is inconsistent [22, 37]. In the SwedeHF registry, the prevalence of all comorbidities was higher in patients with HFpEF, except for CAD, which was more common in those with HFrEF and HFmrEF, reflecting the distinct etiopathogenesis of the different HF phenotypes [13]. Although CAD was not considered among comorbidities in our study, it was one of the most frequent conditions in medical history ranging from 14% in patients with 0–1 to 65% in patients with ≥ 6 comorbidities. Notably, it was shown that ischemic heart disease was associated with a better outcome in our patient cohort, which may be related to improved therapy adherence and advanced revascularization strategies [38].

The most common comorbidity in our registry was arterial hypertension (78%), followed by anemia (51%) and atrial fibrillation/flutter (44%). These findings only partially align with current literature. While arterial hypertension was the most common comorbidity in most HF studies, both in registries [39] and randomized controlled trials [35] as well as in chronic and acute HF [40], the distribution of other comorbidities varies across different studies. The rates of anemia in our patient cohort were significantly higher compared to data from the SwedeHF registry [13], where anemia was present in about one third of HFmrEF patients, and from the Get With The Guidelines (GWTG) registry [41], where only about 20% of HFmrEF patients had anemia. Evidence suggests that anemia is more common in patients with higher LVEF, who usually are older and have more comorbidities [42]. However, our analysis suggests higher rates of anemia in HFmrEF compared to HFmrEF populations with similar baseline characteristics (especially similar mean age and sex distribution) and number of comorbidities [42]. This finding suggests that anemia may be more frequent in patients with HFmrEF than previously described, as specific data for this subgroup is still scarce [43]. Additionally, higher rates of anemia were observed in hospitalized patients, consistent with haemodilution and more severe HF [42]. Further, the role of iron deficiency in HF and its association with anemia is still poorly investigated and may be relevant in understanding the interaction of various comorbidities and prognosis of patients with HFmrEF. Of note, in the present registry iron status was measured in only 27% of patients with HFmrEF and concomitant anemia [43], limiting the ability to assess iron deficiency as a separate comorbidity. On the other hand, rates of atrial fibrillation/flutter were lower compared to data from the SwedeHF registry (44% vs. 58%) [13] but higher than the rates reported in the ESC-HF-Long Term (ESC-HF-LT) registry (44% vs. 22%) [44]. Despite different absolute rates, our findings are consistent with current literature, as atrial fibrillation remains one of the three most frequent comorbidities in patients with HF in various registries [11, 37]. Notably, a comparison between our findings and most current research regarding the distribution of comorbidities is difficult due to the heterogeneity of HF populations included in the different registries or trials.

Consistent with the analysis of the SwedeHF registry, in the present study an increasing number of comorbidities was associated with higher age as well as more severe and symptomatic HF, as reflected by higher New York Heart Association (NYHA) symptom class, higher NT-pro-BNP levels and longer hospitalization time in patients with ≥ 6 comorbidities. Moreover, a higher age is associated with a higher burden of comorbidities [45]. This was confirmed in our study, where the greatest proportion of patients with ≥ 6 comorbidities was found in patients aged ≥ 90 years, whereas most patients aged < 50 years had less than 6 comorbidities. 

The application of first line GDMT of HF (i.e., RAAS-inhibitors, beta-blockers, MRA and SGLT2-inhibitors) in multimorbid patients still represents a challenge in clinical practice, and due to the exclusion of multiple comorbidities from most HF trials the effect of medical therapy according to the comorbidity burden in HF is largely unexplored [12]. Specifically, for the medical treatment of HFmrEF current guidelines do not provide strong recommendations, except for the use of SGLT2-inhibitors which was introduced by a recent update [46]. Therefore, even less data regarding medical treatment and multimorbidity in HFmrEF is available. Evidence suggests that an increase in comorbidities was associated with a lower rate of GDMT prescription in HF patients [12]. In the SwedeHF registry only treatment of HFrEF patients was analysed, however combined use of HF therapy decreased with increasing number of comorbidities, and patients with ≥ 6 comorbidities were less frequently treated with RAAS-inhibitors and beta-blockers, but more commonly received MRA and loop diuretics [13]. Interestingly, our study only partially aligns with these findings, as beta-blockers, MRA and loop diuretics, but not RAAS-inhibitors, were more frequently prescribed for patients with ≥ 6 comorbidities, suggesting that a higher burden of comorbidities in HFmrEF was not associated with lower treatment of GDMT. Notably, the use of SGLT2 inhibitors was more frequent in patients with 4–5 comorbidities, but rates were overall low due to the lack of evidence until end of inclusion for the present registry. Following the new updated guidelines and evidence suggesting beneficial effects of SGLT2 inhibitors even in multimorbidity, the use of SGLT2 inhibitors in HFmrEF is expected to rise [46, 47]. Further, the rates of ARNI use at admission were less than 1% for each comorbidity group. While the rates at discharge were slightly higher in all groups, the overall use of ARNI was generally low. This finding likely reflects the limited recommendation for ARNI use in HFmrEF during the study period, as ARNI therapy was only granted a class IIb recommendation in the 2021 ESC guidelines [8]. Finally, we registered a high rate of ACEi, which may also explain the lower rates of ARNI use. Lower adherence to GDMT in patients with HF and multiple concomitant comorbidities may be related to lower tolerance and higher risk of complications but it might also reflect lack of evidence. The “Safety, Tolerability and Efficacy of Rapid Optimization, Helped by NT-ProBNP Testing, of Heart Failure Therapies” (STRONG-HF) trial demonstrated that the up-titration of HF medical therapy with close follow-up is safe and effective in HF patients regardless of the non-cardiovascular comorbidity burden [26], suggesting a benefit of GDMT in multimorbid HF patients. Optimization of medical treatment of HFmrEF in concomitance of comorbidities is still widely unexplored and while our study suggests a high prevalence of GDMT even in multimorbid patients, further research is needed regarding the associations of medical treatment according to comorbidities and prognosis in HFmrEF.

Multimorbidity in HF has been associated with an adverse outcome in various studies, with proportionally increasing rates of morbidity and mortality alongside the burden of comorbidities. In the present study, patients with ≥ 6 comorbidities had a significantly higher risk of all-cause mortality compared to patients with less comorbidities, which is consistent with data from the SwedeHF registry [13]. Similar findings were reported for the risk of HF rehospitalization, as one third of the patients with ≥ 6 comorbidities was hospitalized for worsening HF during follow-up. Consistent with previous research [18, 19], non-cardiovascular comorbidities had an even greater impact on all-cause mortality than cardiovascular comorbidities and may be a key driver of the higher event rate. When analyzing the prognostic impact of individual comorbidities in our cohort, differences in their association with outcomes were observed. Accordingly, prior or current malignancy, anemia and liver cirrhosis conferred the highest risk for all-cause mortality, while CKD, atrial fibrillation/flutter, and anemia were most strongly associated with HF related rehospitalization. This observation aligns with prior evidence from large HF cohorts, such as the combined PARADIGM-HF and ATMOSPHERE analysis, where the highest individual risk was seen in patients with peripheral artery disease, stroke and anemia, whereas combination of CKD and hypertension had the greatest population-attributable impact [35]. A recent pooled analysis of HFmrEF and HFpEF patients from the “Treatment of Preserved Cardiac Function Heart Failure With an Aldosterone Antagonist” (TOPCAT) and “Prospective Comparison of ARNI with ARB Global Outcomes in HF With Preserved Ejection Fraction” (PARAGON-HF) trials further suggests that specific combinations of comorbidities may synergistically increase event rates [48]. Notably, the comorbidity associated with the highest risk differ depending on the perspective: at the individual level the highest HR was observed for peripheral artery disease and stroke, while at the population level the highest HR was found for the combination of hypertension, diabetes mellitus, and chronic kidney disease [48]. These findings highlight the complex interaction between cardiovascular and non-cardiovascular comorbidities, where certain conditions may have a particularly strong prognostic impact when occurring in specific combinations. Moreover, the findings emphasize the heterogeneity of multimorbidity in HF and suggest that comorbidity profiles rather than simple comorbidity counts may provide a more accurate reflection of risk in HFmrEF patients.

In conclusion, multimorbidity is common in HFmrEF and is associated with poor health outcomes. Stepwise approaches to improve health outcomes in HF such as the ARISE-HF framework and the emphasis of international guidelines on better understanding and managing concomitant comorbidities aim at optimizing treatment of multimorbid HF patients [49]. This study suggests a high burden of comorbidities in HFmrEF patients and highlights the relevant prognostic impact of multimorbidity in HFmrEF, suggesting the need for optimal characterization of HFmrEF patients including concomitant comorbidities. The heterogeneity of HFmrEF populations and the varying adequacy of comorbidity management represent important factors influencing outcomes and should be further addressed in future research. Prospective studies integrating comorbidity severity, treatment adequacy, and refined HFmrEF subgroup stratification may help to better delineate risk profiles and therapeutic implications. Notably, the present findings largely confirm established clinical observations, thereby reinforcing their relevance to real-world practice while underscoring the need for more focused prospective data.

## Limitations

This study has several limitations. Due to the retrospective and single-centre study design, results may be influenced by measured and unmeasured confounding. As this analysis included only hospitalized patients with HFmrEF, selection bias cannot be excluded, and the findings may not be fully generalizable to the overall HFmrEF population. Overall rates of treatment with ARNI and SGLT2i were low, mostly due to lack of evidence for the HFmrEF population including patients until 2022. While this limits the interpretability of therapeutical management in our patient cohort, it also emphasizes the need for future research on the prognostic effects of HF pharmacotherapies in HFmrEF. Comorbidities were considered as fixed at baseline, and potential changes over time were not accounted for. This represents a limitation inherent to the retrospective study design. Detailed information on the clinical severity, stage, or management of individual comorbidities was not available. As all comorbidities were treated as binary variables, potential differences in disease burden or treatment adequacy could not be accounted for and may have influenced outcomes. The predefined set of 12 comorbidities may underestimate the overall multimorbidity burden, as additional relevant conditions were not captured in the present dataset. Due to the low prevalence and incomplete documentation of mental disorders, comorbidities such as dementia and depression that may have influenced patient outcomes could not be included in the present analysis. This underrepresentation likely reflects insufficient systematic recording rather than true absence and should be considered a limitation of our study. Finally, causes of death beyond during index hospitalization were not reported and may have affected the main endpoint of the study.

## Conclusions

The present study investigated the distribution of comorbidities and the prognostic impact of multimorbidity in patients hospitalized with HFmrEF. More than half of all patients presented at least 4 comorbidities, suggestingt multimorbidity is common in HFmrEF patients. The most frequent comorbidities were arterial hypertension, anemia and atrial fibrillation. Furthermore, patients with more comorbidities were older and had more severe HF, and a higher age was associated with a higher burden of comorbidities. Moreover, patients with ≥ 6 comorbidities were more frequently treated with beta-blocker, MRA and loop diuretics. Finally, a higher number of comorbidities was associated with higher risk of all-cause mortality, as well as rehospitalization for worsening HF.

## Supplementary Information

Below is the link to the electronic supplementary material.

ESM 1  Medication at discharge stratified by the number of comorbidities. (PPTX 51.0 KB)

ESM 2 Kaplan-Meier analyses demonstrating the prognostic impact of comorbidities on the risk of all-cause mortality (left panel) and hospitalization for worsening HF (right panel) in patients with HFmrEF stratified by number of comorbidities (i.e., from 0 to 8). (PPTX 44.9 KB)

ESM 3 Forest plots demonstrating the risk of all-cause mortality (left upper panel) and HF-related rehospitalization (left lower panel) stratified by comorbidity groups after multivariable adjustment, as well as the risk of all-cause mortality (right upper panel) and HF-related rehospitalization (right lower panel) for non-cardiovascular vs. cardiovascular comorbidities after multivariable adjustment. (PPTX 106 KB)

ESM 4 (PPTX 55.7 KB)

ESM 5 (DOCX 19.8 KB)

ESM 6 (DOCX 2.43 MB)

ESM 7 (DOCX 3.49 KB)

## Data Availability

The dataset used and/or analysed during the current study are available from the corresponding author upon reasonable request.

## References

[1] Savarese G et al (2023) Global burden of heart failure: a comprehensive and updated review of epidemiology. Cardiovasc Res 118(17):3272–3287 35150240 10.1093/cvr/cvac013

[2] Wong CY et al (2011) Trends in comorbidity, disability, and polypharmacy in heart failure. Am J Med 124(2):136–143 21295193 10.1016/j.amjmed.2010.08.017 PMC3237399

[3] Khan MS et al (2020) Trends in prevalence of comorbidities in heart failure clinical trials. Eur J Heart Fail 22(6):1032–1042 32293090 10.1002/ejhf.1818 PMC7906002

[4] Le Reste JY et al (2013) The European General Practice Research Network presents a comprehensive definition of multimorbidity in family medicine and long term care, following a systematic review of relevant literature. J Am Med Dir Assoc 14(5):319–325 23411065 10.1016/j.jamda.2013.01.001

[5] Barnett K et al (2012) Epidemiology of multimorbidity and implications for health care, research, and medical education: a cross-sectional study. Lancet 380(9836):37–43 22579043 10.1016/S0140-6736(12)60240-2

[6] Salisbury C (2012) Multimorbidity: redesigning health care for people who use it. Lancet 380(9836):7–9 22579042 10.1016/S0140-6736(12)60482-6

[7] Heidenreich PA et al (2022) 2022 AHA/ACC/HFSA guideline for the management of heart failure: a report of the American College of Cardiology/American Heart Association joint committee on clinical practice guidelines. Circulation 145(18):e895–e1032 35363499 10.1161/CIR.0000000000001063

[8] McDonagh TA et al (2022) 2021 ESC Guidelines for the diagnosis and treatment of acute and chronic heart failure: developed by the Task Force for the diagnosis and treatment of acute and chronic heart failure of the European Society of Cardiology (ESC). With the special contribution of the Heart Failure Association (HFA) of the ESC. Eur J Heart Fail 24(1):4–131 10.1002/ejhf.2333 35083827

[9] Wang L et al (2018) A systematic review of cost-of-illness studies of multimorbidity. Appl Health Econ Health Policy 16(1):15–29 28856585 10.1007/s40258-017-0346-6

[10] Larkin J et al (2021) The experience of financial burden for people with multimorbidity: a systematic review of qualitative research. Health Expect 24(2):282–295 33264478 10.1111/hex.13166 PMC8077119

[11] Kuan V et al (2023) Identifying and visualising multimorbidity and comorbidity patterns in patients in the English National Health Service: a population-based study. Lancet Digit Health 5(1):e16–e27 36460578 10.1016/S2589-7500(22)00187-X

[12] Takeuchi S et al (2022) Multimorbidity, guideline-directed medical therapies, and associated outcomes among hospitalized heart failure patients. ESC Heart Fail 9(4):2500–2510 35561100 10.1002/ehf2.13954 PMC9288806

[13] Tomasoni D et al (2024) The role of multimorbidity in patients with heart failure across the left ventricular ejection fraction spectrum: data from the Swedish Heart Failure Registry. Eur J Heart Fail 26(4):854–868 38131248 10.1002/ejhf.3112

[14] Tisminetzky M et al (2018) Multimorbidity burden and adverse outcomes in a community-based cohort of adults with heart failure. J Am Geriatr Soc 66(12):2305–2313 30246862 10.1111/jgs.15590 PMC6697073

[15] Gracia Gutiérrez A et al (2024) Multimorbidity in incident heart failure: characterisation and impact on 1-year outcomes. J Clin Med. 10.3390/jcm13133979 38999543 10.3390/jcm13133979 PMC11242217

[16] Conrad N et al (2018) Temporal trends and patterns in heart failure incidence: a population-based study of 4 million individuals. Lancet 391(10120):572–580 29174292 10.1016/S0140-6736(17)32520-5 PMC5814791

[17] Lee KS et al (2023) Relationship between comorbidity and health outcomes in patients with heart failure: a systematic review and meta-analysis. BMC Cardiovasc Disord 23(1):498 37817062 10.1186/s12872-023-03527-x PMC10563307

[18] Manemann SM et al (2016) Multimorbidity in heart failure: effect on outcomes. J Am Geriatr Soc 64(7):1469–1474 27348135 10.1111/jgs.14206 PMC4943753

[19] Correale M et al (2021) Non-cardiovascular comorbidities in heart failure patients and their impact on prognosis. Kardiol Pol 79(5):493–502 34125921 10.33963/KP.15934

[20] Lewis EF (2013) Assessing the impact of heart failure therapeutics on quality of life and functional capacity. Curr Treat Options Cardiovasc Med 15(4):425–436 23625508 10.1007/s11936-013-0249-2

[21] Rushton CA, Kadam UT (2014) Impact of non-cardiovascular disease comorbidity on cardiovascular disease symptom severity: a population-based study. Int J Cardiol 175(1):154–161 24856803 10.1016/j.ijcard.2014.05.001 PMC4078220

[22] Streng KW et al (2018) Non-cardiac comorbidities in heart failure with reduced, mid-range and preserved ejection fraction. Int J Cardiol 271:132–139 30482453 10.1016/j.ijcard.2018.04.001

[23] van Deursen VM et al (2014) Co-morbidities in patients with heart failure: an analysis of the European Heart Failure Pilot Survey. Eur J Heart Fail 16(1):103–111 24453099 10.1002/ejhf.30

[24] Chioncel O et al (2023) Comprehensive characterization of non-cardiac comorbidities in acute heart failure: an analysis of ESC-HFA EURObservational Research Programme Heart Failure Long-Term Registry. Eur J Prev Cardiol 30(13):1346–1358 37172316 10.1093/eurjpc/zwad151

[25] Bhatt AS et al (2020) The burden of non-cardiac comorbidities and association with clinical outcomes in an acute heart failure trial - insights from ASCEND-HF. Eur J Heart Fail 22(6):1022–1031 32212297 10.1002/ejhf.1795 PMC7394726

[26] Chioncel O et al (2023) Non-cardiac comorbidities and intensive up-titration of oral treatment in patients recently hospitalized for heart failure: insights from the STRONG-HF trial. Eur J Heart Fail 25(11):1994–2006 37728038 10.1002/ejhf.3039

[27] Chamberlain AM et al (2015) Multimorbidity in heart failure: a community perspective. Am J Med 128(1):38–45 25220613 10.1016/j.amjmed.2014.08.024 PMC4282820

[28] Stolfo D et al (2023) Heart failure pharmacological treatments and outcomes in heart failure with mildly reduced ejection fraction. Eur Heart J 9(6):526–535 10.1093/ehjcvp/pvad036 PMC10509568 37204037

[29] Savarese G et al (2020) Comorbidities and cause-specific outcomes in heart failure across the ejection fraction spectrum: a blueprint for clinical trial design. Int J Cardiol 313:76–82 32360702 10.1016/j.ijcard.2020.04.068

[30] Schupp T et al (2024) Prevalence and prognosis of aortic valve diseases in patients hospitalized with heart failure with mildly reduced ejection fraction. Eur J Heart Fail. 10.1002/ejhf.3337 38896059 10.1002/ejhf.3337

[31] Abel N et al (2024) Left ventricular diastolic dysfunction in patients with heart failure with mildly reduced ejection fraction. Int J Cardiol 414:132386 39079587 10.1016/j.ijcard.2024.132386

[32] Popescu BA et al (2009) European Association of Echocardiography recommendations for training, competence, and quality improvement in echocardiography. Eur J Echocardiogr 10(8):893–905 19889658 10.1093/ejechocard/jep151

[33] Vestbo J et al (2013) Global strategy for the diagnosis, management, and prevention of chronic obstructive pulmonary disease: GOLD executive summary. Am J Respir Crit Care Med 187(4):347–365 22878278 10.1164/rccm.201204-0596PP

[34] Tromp J et al (2018) Multimorbidity in patients with heart failure from 11 Asian regions: a prospective cohort study using the ASIAN-HF registry. PLoS Med 15(3):e1002541 29584721 10.1371/journal.pmed.1002541 PMC5870945

[35] Dewan P et al (2023) Impact of multimorbidity on mortality in heart failure with reduced ejection fraction: which comorbidities matter most? An analysis of PARADIGM-HF and ATMOSPHERE. Eur J Heart Fail 25(5):687–697 37062869 10.1002/ejhf.2856

[36] Yang M et al (2023) Impact of comorbidities on health status measured using the Kansas City Cardiomyopathy Questionnaire in patients with heart failure with reduced and preserved ejection fraction. Eur J Heart Fail 25(9):1606–1618 37401511 10.1002/ejhf.2962

[37] Savarese G et al (2022) Heart failure with mid-range or mildly reduced ejection fraction. Nat Rev Cardiol 19(2):100–116 34489589 10.1038/s41569-021-00605-5 PMC8420965

[38] Schupp T et al (2024) Distribution and prognostic impact of different heart failure etiologies in patients with heart failure with mildly reduced ejection fraction. Eur J Intern Med 130:86–97 39030147 10.1016/j.ejim.2024.07.009

[39] Bánfi-Bacsárdi F et al (2024) Therapeutic consequences and prognostic impact of multimorbidity in heart failure: time to act. J Clin Med. 10.3390/jcm14010139 39797222 10.3390/jcm14010139 PMC11722306

[40] Crespo-Leiro MG et al (2016) European Society of Cardiology Heart Failure Long-Term Registry (ESC-HF-LT): 1-year follow-up outcomes and differences across regions. Eur J Heart Fail 18(6):613–625 27324686 10.1002/ejhf.566

[41] Shah KS et al (2017) Heart failure with preserved, borderline, and reduced ejection fraction: 5-year outcomes. J Am Coll Cardiol 70(20):2476–2486 29141781 10.1016/j.jacc.2017.08.074

[42] Savarese G et al (2020) Prevalence of, associations with, and prognostic role of anemia in heart failure across the ejection fraction spectrum. Int J Cardiol 298:59–65 31521440 10.1016/j.ijcard.2019.08.049

[43] Schupp T et al (2024) Effect of anaemia and iron deficiency in heart failure with mildly reduced ejection fraction. Eur J Clin Invest 54(8):e14205 38597298 10.1111/eci.14205

[44] Chioncel O et al (2017) Epidemiology and one-year outcomes in patients with chronic heart failure and preserved, mid-range and reduced ejection fraction: an analysis of the ESC Heart Failure Long-Term Registry. Eur J Heart Fail 19(12):1574–1585 28386917 10.1002/ejhf.813

[45] Regan JA et al (2019) Impact of age on comorbidities and outcomes in heart failure with reduced ejection fraction. JACC: Heart Failure 7(12):1056–1065 31779928 10.1016/j.jchf.2019.09.004 PMC6936107

[46] McDonagh TA et al (2024) 2023 Focused Update of the 2021 ESC Guidelines for the diagnosis and treatment of acute and chronic heart failure: Developed by the task force for the diagnosis and treatment of acute and chronic heart failure of the European Society of Cardiology (ESC) With the special contribution of the Heart Failure Association (HFA) of the ESC. Eur J Heart Fail 26(1):5–17 38169072 10.1002/ejhf.3024

[47] Rodil Fraile R, Malafarina V, Tiberio López G (2018) Sacubitril-valsartan in heart failure and multimorbidity patients. ESC Heart Fail 5(5):956–959 30039930 10.1002/ehf2.12338 PMC6165940

[48] Yang M et al (2025) Impact of multimorbidity on mortality in heart failure with mildly reduced and preserved ejection fraction. Circ Heart Fail 18(3):e011598 40026147 10.1161/CIRCHEARTFAILURE.124.011598

[49] Stewart S et al (2016) Establishing a pragmatic framework to optimise health outcomes in heart failure and multimorbidity (ARISE-HF): a multidisciplinary position statement. Int J Cardiol 212:1–10 27015641 10.1016/j.ijcard.2016.03.001 PMC5646657

