# Supplementary material for: Prevalence and prognosis of multimorbidity in heart failure with mildly reduced ejection fraction
Source: Clin Res Cardiol. 2026 Jan 19;115(9):1615–28. doi: 10.1007/s00392-025-02840-z (PMC13457439; doi:10.1007/s00392-025-02840-z)
Supplement: Supplementary file 1 [file 392_2025_2840_MOESM5_ESM.docx]

| **Supplementary table 1. Baseline characteristics of the overall HARMER-Registry population** | | |
| --- | --- | --- |
| **Characteristics** | **HARMER-Registry**  (*n* = 2.184) | |
| **Age**, Median (IQR) | 76 (64-83) | |
| **Male sex**, n (%) | 1410 | (64.6) |
| **Body-Mass-Index,** kg/m^2^, Median (IQR) | 26.6 (23.8-30.7) | |
| **Systolic blood pressure**, mmHg, Median (IQR) | 142 (125-163) | |
| **Diastolic blood pressure**, mmHg, Median (IQR) | 79 (69-90) | |
| **Heart rate**, bpm, Median (IQR) | 80 (68-95) | |
| **Medical history**, n (%) |  |  |
| Coronary artery disease | 896 | (41.1) |
| Acute myocardial infarction | 521 | (23.9) |
| PCI | 612 | (28.0) |
| CABG | 214 | (9.8) |
| Prior valvular surgery | 96 | (4.4) |
| Prior congestive heart failure | 741 | (33.9) |
| Decompensated heart failure < 12 months | 238 | (10.9) |
| Prior ICD | 42 | (1.9) |
| Prior sICD | 9 | (0.4) |
| Prior CRT-D | 32 | (1.5) |
| Prior Pacemaker | 200 | (9.2) |
| Chronic kidney disease | 679 | (31.1) |
| Peripheral artery disease | 253 | (11.6) |
| Stroke | 331 | (15.2) |
| Liver cirrhosis | 47 | (2.2) |
| Malignancy | 335 | (15.3) |
| COPD | 263 | (12.0) |
| **Cardiovascular risk factors,** n (%) |  |  |
| Arterial hypertension | 1702 | (77.9) |
| Diabetes mellitus | 799 | (36.6) |
| Hyperlipidemia | 662 | (30.3) |
| Smoking |  | |
| Current | 406 | (18.6) |
| Former | 390 | (17.9) |
| Family history | 201 | (9.2) |
| **Comorbidities at index hospitalization**,  n (%) |  |  |
| STEMI | 176 | (8.1) |
| NSTEMI | 274 | (12.5) |
| Acute decompensated heart failure | 484 | (22.2) |
| Cardiogenic shock | 53 | (2.4) |
| Cardiopulmonary resuscitation | 53 | (2.4) |
| Out-of-hospital | 22 | (1.0) |
| In-hospital | 31 | (1.4) |
| Stroke | 298 | (13.6) |
| CABG, coronary artery bypass grafting; COPD, chronic obstructive pulmonary disease; CRT-D, cardiac resynchronization therapy with defibrillator; ICD, implantable cardioverter defibrillator; IQR, interquartile range; (N)STEMI, non-ST-segment elevation myocardial infarction; s-ICD, subcutaneous implantable cardioverter defibrillator.  Level of significance p≤0.05. Bold type indicates statistical significance. | | |
