# Supplementary material for: Prevalence and prognosis of multimorbidity in heart failure with mildly reduced ejection fraction
Source: Clin Res Cardiol. 2026 Jan 19;115(9):1615–28. doi: 10.1007/s00392-025-02840-z (PMC13457439; doi:10.1007/s00392-025-02840-z)
Supplement: Supplementary file 2 [file 392_2025_2840_MOESM6_ESM.docx]

| **Supplementary table 2. Heart-failure related and procedural data.** | | | | | | | | | |
| --- | --- | --- | --- | --- | --- | --- | --- | --- | --- |
|  | **0-1**  **Comorbidities**  (*n*=241) | | **2-3 Comorbidities**  (*n*=765) | | **4-5**  **Comorbidities**  (*n*=800) | | **≥6**  **Comorbidities**  (*n*=378) | | **p value** |
| **Heart failure etiology**, n (%) |  |  |  |  |  |  |  |  |  |
| Ischemic cardiomyopathy | 93 | (38.6) | 412 | (53.9) | 475 | (59.4) | 278 | (73.5) | **0.001** |
| Non-ischemic cardiomyopathy | 32 | (13.3) | 52 | (6.8) | 41 | (5.1) | 24 | (6.3) |  |
| Hypertensive cardiomyopathy | 18 | (7.5) | 87 | (11.4) | 56 | (7.0) | 17 | (4.5) |  |
| Congenital heart disease | 1 | (0.4) | 2 | (0.3) | 1 | (0.1) | 0 | (0.0) |  |
| Valvular heart disease | 8 | (3.3) | 23 | (3.0) | 45 | (5.6) | 20 | (5.3) |  |
| Tachycardia-associated | 12 | (5.4) | 54 | (6.6) | 51 | (6.4) | 11 | (3.2) |  |
| Tachymyopathy | 8 | (3.3) | 16 | (2.1) | 12 | (1.5) | 2 | (0.5) |  |
| Pacemaker-induced cardiomyopathy | 1 | (0.4) | 8 | (1.0) | 8 | (1.0) | 2 | (0.5) |  |
| Unknown | 76 | (31.5) | 127 | (16.6) | 123 | (15.4) | 26 | (6.9) |  |
| **NYHA functional class,** n (%) |  |  |  |  |  |  |  |  |  |
| I/II | 214 | (95.5) | 627 | (76.1) | 549 | (69.1) | 195 | (57.0) | **0.001** |
| III | 21 | (8.7) | 98 | (12.8) | 168 | (21.0) | 123 | (32.5) |  |
| IV | 6 | (2.5) | 40 | (5.2) | 83 | (10.4) | 60 | (15.9) |  |
| **Echocardiographic data** |  |  |  |  |  |  |  |  |  |
| LVEF, %, median (IQR) | 45 (45-47) | | 45 (45-47) | | 45 (45-47) | | 45 (45-46) | | **0.009** |
| IVSd, mm, median (IQR) | 11 (10-12) | | 12 (11-13) | | 12 (11-13) | | 12 (11-13) | | **0.001** |
| LVEDD, mm, median (IQR) | 49 (45-53) | | 49 (44-54) | | 48 (44-54) | | 50 (45-54) | | 0.154 |
| TAPSE, mm, median (IQR) | 21 (19-24) | | 20 (18-23) | | 20 (17-23) | | 19 (16-22) | | **0.001** |
| LA diameter, mm, median (IQR) | 37 (33-41) | | 41 (36-46) | | 43 (38-48) | | 46 (39-50) | | **0.001** |
| LA area, cm^2^, median (IQR) | 17 (15-20) | | 20 (16-24) | | 23 (19-27) | | 25 (20-29) | | **0.001** |
| E/A, median (IQR) | 0.9 (0.7-1.2) | | 0.8 (0.6-1.1) | | 0.8 (0.6-1.2) | | 1.0 (0.6-1.5) | | **0.001** |
| E/E`, median (IQR) | 7.1 (5.5-10.0) | | 9.0 (5.8-12.6) | | 10.5 (7.5-14.5) | | 12.0 (7.5-17.0) | | **0.001** |
| Diastolic dysfunction, n (%) | 142 | (58.9) | 555 | (72.5) | 601 | (75.1) | 276 | (73.0) | **0.001** |
| Moderate-severe aortic stenosis, n (%) | 2 | (0.8) | 26 | (4.3) | 113 | (14.1) | 73 | (19.3) | **0.001** |
| Moderate-severe aortic regurgitation, n (%) | 3 | (1.2) | 14 | (1.8) | 35 | (4.4) | 32 | (8.5) | **0.001** |
| Moderate-severe mitral regurgitation, n (%) | 4 | (1.7) | 46 | (6.0) | 116 | (14.5) | 96 | (25.4) | **0.001** |
| Moderate-severe tricuspid regurgitation, n (%) | 4 | (1.7) | 45 | (5.9) | 150 | (18.8) | 145 | (38.4) | **0.001** |
| **Coronary angiography,** n (%) | 118 | (49.0) | 378 | (49.4) | 283 | (35.4) | 121 | (32.0) | **0.001** |
| No evidence of coronary artery disease | 41 | (34.7) | 76 | (20.1) | 47 | (16.6) | 11 | (9.1) | **0.001** |
| 1-vessel disease | 28 | (23.7) | 74 | (19.6) | 51 | (18.0) | 13 | (10.7) |  |
| 2-vessel disease | 27 | (22.9) | 91 | (24.1) | 53 | (18.7) | 21 | (17.4) |  |
| 3-vessel disease | 22 | (18.6) | 137 | (36.2) | 132 | (46.6) | 76 | (62.8) |  |
| CABG | 1 | (0.8) | 14 | (3.7) | 38 | (13.4) | 20 | (16.5) | **0.001** |
| Chronic total occlusion | 11 | (9.3) | 39 | (10.3) | 41 | (14.5) | 22 | (18.2) | 0.063 |
| PCI, n (%) | 64 | (54.2) | 208 | (55.0) | 144 | (50.9) | 65 | (53.7) | 0.763 |
| Sent to CABG, n (%) | 3 | (2.5) | 25 | (6.6) | 19 | (6.7) | 4 | (3.3) | 0.201 |
| **Baseline laboratory values**, median (IQR) |  |  |  |  |  |  |  |  |  |
| Potassium, mmol/L | 3.9 (3.6-4.1) | | 3.9 (3.6-4.1) | | 3.9 (3.6-4.2) | | 3.9 (3.6-4.3) | | 0.181 |
| Sodium, mmol/L | 140 (138-141) | | 139 (137-141) | | 139 (137-142) | | 139 (137-142) | | 0.283 |
| Creatinine, mg/dL | 0.89 (0.76-1.03) | | 0.96 (0.81-1.17) | | 1.15 (0.92-1.53) | | 1.62 (1.20-2.28) | | **0.001** |
| eGFR, mL/min/1.73 m^2^ | 89 (74-101) | | 76 (59-93) | | 58 (42-76) | | 40 (27-55) | | **0.001** |
| Hemoglobin, g/dL | 14.2 (13.2-15.2) | | 13.3 (11.8-14.5) | | 11.7 (10.0-13.2) | | 10.5 (9.3-11.8) | | **0.001** |
| WBC count, x 10^9^/L | 8.47 (6.45-10.09) | | 8.28 (6.48-9.99) | | 7.98 (6.37-10.16) | | 8.21 (6.61-10.32) | | 0.415 |
| Platelet count, x 10^9^/L | 238 (196-283) | | 231 (185-285) | | 222 (172-287) | | 222 (167-286) | | **0.008** |
| HbA1c, % | 5.5 (5.2-5.8) | | 5.8 (5.5-6.4) | | 6.1 (5.6-7.1) | | 6.5 (5.8-7.6) | | **0.001** |
| LDL- cholesterol, mg/L | 120 (95-149) | | 104 (81-132) | | 91 (70-121) | | 77 (62-101) | | **0.001** |
| HDL- cholesterol, mg/L | 45 (37-54) | | 43 (35-53) | | 41 (34-51) | | 39 (31-47) | | **0.001** |
| C-reactive protein, mg/L | 6 (3-25) | | 9 (3-34) | | 15 (5-48) | | 27 (9-67) | | **0.001** |
| NT-pro BNP, pg/mL | 735 (169-2655) | | 1590 (484-3291) | | 3758 (1691-8296) | | 6410 (2667-13157) | | **0.001** |
| NT-pro BNP (eGFR corrected), pg/mL | 1062 (227-2990) | | 1206 (371-2580) | | 1868 (833-4009) | | 2316 (1139-5081) | | **0.001** |
| Cardiac troponin I, µg/L | 0.02 (0.02-0.28) | | 0.02 (0.02-0.21) | | 0.03 (0.02-0.13) | | 0.06 (0.02-0.21) | | **0.001** |
| CABG, coronary artery bypass grafting; eGFR, estimated glomerular filtration rate; HbA1c, glycated hemoglobin; HDL, high-density lipoprotein; IQR, interquartile range; IVSd, Interventricular septal end diastole; LA, left atrial; LDL, low-density lipoprotein; LVEDD, Left ventricular end-diastolic diameter; LVEF, left ventricular ejection fraction; NT-pro BNP, aminoterminal pro-B-type natriuretic peptide; NYHA, New York Heart Association; PCI, percutaneous coronary intervention; TAPSE, tricuspid annular plane systolic excursion; WBC, white blood cells.  Level of significance p≤0.05. Bold type indicates statistical significance. | | | | | | | | | |
