# Supplementary material for: Prevalence and prognosis of multimorbidity in heart failure with mildly reduced ejection fraction
Source: Clin Res Cardiol. 2026 Jan 19;115(9):1615–28. doi: 10.1007/s00392-025-02840-z (PMC13457439; doi:10.1007/s00392-025-02840-z)
Supplement: Supplementary file 3 [file 392_2025_2840_MOESM7_ESM.docx]

| **Supplementary table 3.** **Distribution of the number of comorbidities stratified by age.** | | | | | | | | |  |
| --- | --- | --- | --- | --- | --- | --- | --- | --- | --- |
|  | **0-1 comorbidities**  (*n*=241) | | **2-3 comorbidities** (*n*=765) | | **4-5 comorbidities**  (*n*=800) | | **≥6 comorbidities**  (*n*=378) | |  |
| **Age (years),** n (%) |  | |  | |  | |  | |  |
| <20 | 2 | (100.0) | 0 | (0.0) | 0 | (0.0) | 0 | (0.0) | |
| 20-29 | 9 | (60.0) | 6 | (40.0) | 0 | (0.0) | 0 | (0.0) | |
| 30-39 | 27 | (60.0) | 17 | (37.8) | 1 | (2.2) | 0 | (0.0) | |
| 40-49 | 43 | (47.8) | 40 | (44.4) | 7 | (7.8) | 0 | (0.0) | |
| 50-59 | 59 | (24.5) | 120 | (49.8) | 53 | (22.0) | 9 | (3.7) | |
| 60-69 | 41 | (10.1) | 180 | (44.3) | 140 | (34.5) | 45 | (11.1) | |
| 70-79 | 34 | (5.4) | 222 | (35.2) | 245 | (38.8) | 130 | (20.6) | |
| 80-89 | 23 | (3.6) | 163 | (25.2) | 298 | (46.2) | 161 | (25.0) | |
| ≥90 | 3 | (2.8) | 17 | (15.6) | 56 | (51.4) | 33 | (30.3) | |
| Level of significance p≤0.05. Bold type indicates statistical significance. | | | | | | | | | |
